# International Classification of Diseases-10th Revision Surrogates of the Modified Frailty Index and 12-Month Referral to the Hospital in an Older Population from Germany

**DOI:** 10.3390/jcm12237290

**Published:** 2023-11-24

**Authors:** Karel Kostev, Verena Altmann, Josep Maria Haro, Ai Koyanagi, Christian Tanislav, Razak M. Gyasi, Louis Jacob

**Affiliations:** 1 Epidemiology, IQVIA, 60549 Frankfurt, Germany; 2 University Hospital, Philipps University Marburg, 35037 Marburg, Germany; 3 Research and Development Unit, Parc Sanitari Sant Joan de Déu, CIBERSAM, ISCIII, Dr. Antoni Pujadas, 42, Sant Boi de Llobregat, 08830 Barcelona, Spain; josepmaria.haro@sjd.es (J.M.H.);; 4 Department of Geriatrics and Neurology, Diakonie Hospital Jung Stilling, 57074 Siegen, Germany; 5 African Population and Health Research Center, Nairobi 00100, Kenya; 6 National Centre for Naturopathic Medicine, Faculty of Health, Southern Cross University, Lismore 2480, Australia; 7 Université Paris Cité, AP-HP, Lariboisière-Fernand Widal Hospital, Department of Physical Medicine and Rehabilitation, 75010 Paris, France; 8 Université Paris Cité, Inserm U1153, Epidemiology of Ageing and Neurodegenerative Diseases (EpiAgeing), 75010 Paris, France

**Keywords:** ICD-10 surrogates, modified frailty index, mFI, hospital referral, older population, Germany

## Abstract

*Background:* The International Classification of Diseases-10th revision (ICD-10) surrogates of the modified frailty index (mFI) have been defined in recent research. This study aimed to investigate the prevalence of these ICD-10 surrogates and their association with hospital referral in an older population from Germany. *Methods:* The present sample included adults aged ≥65 years followed in German general practices between 2010 and 2021. The index date was the most recent visit date. There were 11 ICD-10 surrogates of the mFI, including a total of 52 diagnoses. These surrogates were assessed in the 12 months prior to the index date. Referral to the hospital was analyzed in the 12 months following the index date. Covariates included age and sex. *Results:* There were 1,406,038 patients included in the study (mean (standard deviation) age 77.0 (7.9) years; 56.2% women). The prevalence of the ICD-10 surrogates of the mFI ranged from 3.3% for a “history of transient ischemic attack or stroke without neurological deficit” to 68.1% for a “history of hypertension requiring medication”. In addition, 24%, 37%, and 23% of participants had 1, 2–3, and >3 ICD-10 surrogates of the mFI, respectively. There was a positive and significant relationship between the number of ICD-10 surrogates and 12-month incident hospital referral (reference: 0 surrogate; 1 surrogate: HR = 1.37, 95% CI = 1.31–1.42; 2–3 surrogates: HR = 2.00, 95% CI = 1.93–2.08; >3 surrogates: HR = 3.32, 95% CI = 3.19–3.44). *Conclusions:* ICD-10 surrogates of the mFI were relatively frequent and were significantly associated with 12-month incident hospital referral in this sample of older adults from general practices in Germany.

## 1. Introduction

Frailty corresponds to a syndrome defined by increased vulnerability to endogenous and exogenous stressors [1]. The prevalence of frailty ranges from 12% to 24% in the worldwide older population [2]. Given that modern societies are aging [3], it is likely that the number of people with frailty will increase in the future. Frailty is a risk factor for several chronic conditions (e.g., cardiovascular disease [4] and dementia [5]), disability [6], and all-cause mortality [7]. Moreover, frailty is associated with a substantial economic burden and leads to an increase in healthcare costs. These relationships likely involve an increased number of general practice consultations, more hospital admissions, and longer inpatient stays [8,9]. Taking these facts together, there is an urgent need to better understand the epidemiology of frailty in older adults.

Frailty is a phenomenon that is complex to assess. Several measures of this syndrome have been developed in the past decades. In the International Classification of Diseases-10th revision (ICD-10), there is no code for frailty apart from R54 (i.e., age-related physical debility or senility) [10]. Moreover, frailty is rarely documented in primary care practices. One of these measures is the 70-item Frailty Index. This measure was used for the first time in a sample of older people from Canada in the early 2000s [11,12]. A modified version of the Frailty Index, also known as the modified Frailty Index (mFI), was later identified [13]. The mFI was found to be a valid predictor of morbidity and mortality in around 1900 patients who had undergone lobectomy. The mFI is based on 11 variables, offering the advantage of assessing frailty with fewer items than with the original index. Other research showed that the mFI also predicts in-hospital mortality and length of stay in an intensive care unit setting [14]. Interestingly, more recently, a study identified 11 surrogates of the mFI based on the ICD-10 [15]. These 11 ICD-10 surrogates include 54 different diagnoses. These surrogates allow the possible use of administrative databases and electronic records to investigate frailty with the mFI. However, to the best of the authors’ knowledge, no research has yet shown how frequent the ICD-10 surrogates of the mFI are in primary care settings. Moreover, it is unknown whether these surrogates significantly predict future hospitalizations.

Therefore, the goal of this study was to investigate the prevalence of ICD-10 surrogates of the mFI and their association with 12-month referral to the hospital in older adults followed in general practices in Germany. The first hypothesis was that the ICD-10 surrogates of the mFI were relatively frequent in this population. The second hypothesis was that the odds of 12-month referral to the hospital increased with the number of surrogates. Given that general practices play a key role in the management of older people, it is of utmost importance to better characterize the epidemiology of frailty in this setting.

## 2. Methods

### 2.1. Database

The present study used data from the Disease Analyzer database (IQVIA). This database has already been described in the literature [16]. Briefly, the Disease Analyzer database contains demographic, diagnosis, and prescription data collected in general and specialized practices in Germany. The data are transferred from the computer systems of the practices to IQVIA every month. Diagnoses are coded using the ICD-10, while prescriptions are coded using the Anatomical Classification of Pharmaceutical Products of the European Pharmaceutical Market Research Association (EphMRA). The quality of the data is regularly assessed based on several criteria, such as the linkage between diagnoses and prescriptions and completeness of the information. General and specialized practices included in the database are selected based on statistics published by the German Medical Association every year. These statistics provide data on multiple variables (e.g., age of the physician, specialty, community size category, and German federal state). Finally, the Disease Analyzer database includes around 3% of all practices in Germany, while previous research has shown that the database is representative of the country.

### 2.2. Study Population

There were 9,450,118 patients followed in 1284 general practices in Germany between 2010 and 2021. The index date corresponded to the most recent visit during the study period. To be included in the study, individuals had to be aged ≥65 years at the index date and be followed for at least 12 months prior to the index date.

### 2.3. ICD-10 Surrogates of the mFI

The 11 ICD-10 surrogates of the mFI and their respective codes are displayed in Table 1. The number of ICD-10 surrogates or the mFI was included in the analyses as a four-category variable (i.e., zero, one, two to three, and more than three surrogates). Following previous research using the original mFI [17], frailty corresponded to the presence of more than three ICD-10 surrogates of the mFI. Pre-frailty corresponded to the presence of one or two to three surrogates. In some analyses, the number of ICD-10 surrogates of the mFI was included as a three-category (i.e., zero, one to two, and at least three surrogates) or a continuous variable.

### 2.4. Statistical Analyses

The demographic characteristics of study patients are described using mean (standard deviation) for continuous variables and N (%) for categorical variables. The prevalence of the 11 ICD-10 surrogates of the mFI was studied in the overall sample and age (i.e., 65–69, 70–74, 75–79, 80–84, 85–89, and ≥90 years) and sex groups (i.e., female and male). The proportion of people with zero, one, two to three, and more than three ICD-10 surrogates was further analyzed in the whole population and by age and sex. Finally, the association between the number of ICD-10 surrogates of the mFI and 12-month referral to the hospital was investigated in the overall sample and age and sex groups using Cox regression models. Models were adjusted for age and sex in the analyses based on the whole population, sex in the age-stratified analyses, and age in the sex-stratified analyses. Sensitivity analyses were conducted in the overall sample with the number of ICD-10 surrogates of the mFI included in the regression models as a three-category variable, as in a previous publication [14] (i.e., zero, one to two, and at least three surrogates), and also as a continuous variable. The results of the Cox regression models are displayed as hazard ratios (HRs) and 95% confidence intervals (CIs). P-values lower than 0.050 were considered statistically significant. Analyses were performed using SAS 9.4 (The SAS Institute, Cary, NC, USA).

## 3. Results

**Figure 1:**
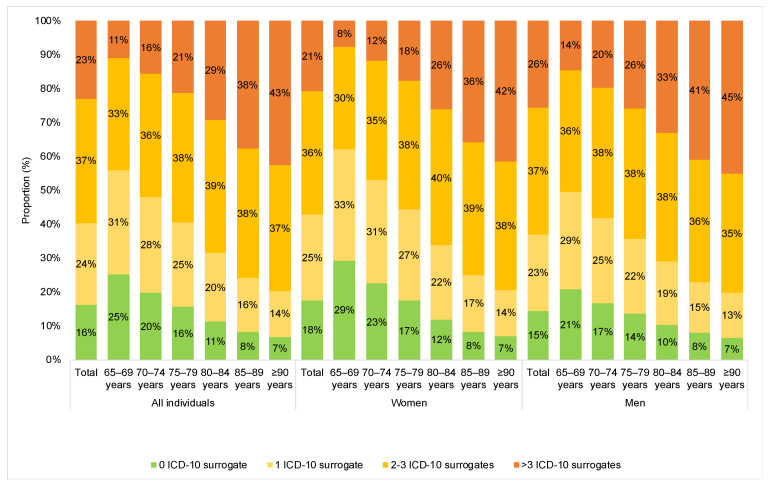
Proportions of individuals with zero, one, two to three, and more than three ICD-10 surrogates of the modified Frailty Index in the overall sample and by age and sex. Abbreviation: ICD-10, International Classification of Diseases-10th revision.

This study included 1,406,038 patients. The mean (standard deviation) age was 77.0 (7.9) years, while the proportion of women was 56.2% (Table 2). The prevalence of the 11 ICD-10 surrogates of the mFI ranged from 3.3% for surrogate 9 “history of TIA or stroke without neurological deficit” to 68.1% for surrogate 2 “history of hypertension requiring medication” (Table 3). The respective figures were 3.2% and 68.0% in women (Supplementary Table S1) and 3.5% and 68.2% in men (Supplementary Table S2). The proportion of people with one, two to three, and more than three ICD-10 surrogates of the mFI was 24%, 37%, and 23% in the overall population (Figure 1). The proportion of individuals with more than three ICD-10 surrogates (i.e., with frailty) increased with age from 11% in those aged 65–69 years to 43% in those aged ≥90 years. A similar trend in the prevalence of more than three ICD-10 surrogates was observed in women and men (women: 8% in the age group 65–69 years versus 42% in the age group ≥90 years; men: 14% in the age group 65-69 years versus 45% in the age group ≥90 years). The results of the adjusted Cox regression models are displayed in Table 4. In the overall sample, compared with zero ICD-10 surrogate of the mFI, one (HR = 1.37, 95% CI = 1.31–1.42), two to three (HR = 2.00, 95% CI = 1.93–2.08), and more than three surrogates (HR = 3.32, 95% CI = 3.19–3.44) were positively and significantly associated with 12-month incident hospital referral. There was also a graded and statistically significant relationship between the number of ICD-10 surrogates of the mFI and 12-month incident hospital referral in all age and sex groups. Finally, the sensitivity analyses corroborated the results, and one to two (HR = 1.58, 95% CI = 1.52–1.64) and at least three surrogates (HR = 2.82, 95% CI = 2.72–2.93) were associated with an increased risk of incident hospital referral compared with zero surrogate (data only shown in the text). Similar findings were obtained when the number of ICD-10 surrogates of the mFI was included in the Cox regression model as a continuous variable (per one-surrogate increase: HR = 1.24, 95% CI = 1.23–1.24).

## 4. Discussion

### 4.1. Main Findings

In this retrospective study, including 1,406,038 older adults from Germany, 61% of the participants had one or two to three ICD-10 surrogates of the mFI (i.e., had pre-frailty), and 23% had more than three surrogates (i.e., had frailty). The prevalence of more than three ICD-10 surrogates increased from 11% in people aged 65–69 years to 43% in those aged ≥90 years. Finally, there was a statistically significant association between the number of ICD-10 surrogates of the mFI and 12-month referral to the hospital, with HR ranging from 1.37 for one surrogate to 3.32 for more than three surrogates (reference: zero surrogate). To the best of the authors’ knowledge, this study is the first to investigate the prevalence of ICD-10 surrogates of the mFI and the relationship between the number of surrogates and hospital referral in the older population followed in primary care practices.

### 4.2. Interpretation of the Findings

A critical finding of this study is that 23% of older adults had more than three ICD-10 surrogates of the mFI and displayed frailty. In addition, the proportion of people with one or two to three surrogates (i.e., with pre-frailty) was 61%. These results are in line with the scientific literature showing that pre-frailty and frailty are common in old age in Germany. For example, a systematic review and meta-analysis of 12 studies revealed that the prevalence of frailty in the older German population ranged from 2.4% to 25.6%, while the pooled prevalence of pre-frailty was 40.2% [18]. Interestingly, in the present study, the proportion of individuals with more than three ICD-10 surrogates of the mFI increased with age. This finding corroborates previous research conducted in Europe. In a cohort of 5632 individuals living in Italy, the prevalence of frailty and pre-frailty increased from 65–69 years to ≥ 80 years at three different time points (e.g., from 1.1% to 12.8% for frailty and from 37.7% to 55.6% for pre-frailty at T0) [19]. At the international level, a study of 60,816 participants from 18 European countries further revealed that there was an increase in the prevalence of frailty from 3.0% to 32.8% and pre-frailty from 38.7% to 52.3% between the age groups 50–64 and ≥85 years [20].

Another major study result is the positive and significant association between the number of ICD-10 surrogates of the mFI and 12-month referral to the hospital. This relationship was statistically significant when the number of surrogates was included in the Cox regression models either as a categorical or a continuous variable. In a cohort of 19,114 older adults from Australia and the USA, frailty and pre-frailty, which were assessed using a modified Fried phenotype and a deficit accumulation Frailty Index, were associated with an increased risk of incident hospitalization for heart failure [4]. A Canadian retrospective cohort study further showed, in a sample of 228,679 home care patients predominantly aged 60 years or over, that frailty significantly predicted hospital admission in the following 90 days [21].

There are several hypotheses to explain the longitudinal association between frailty and referral to the hospital. First, some data suggest a bidirectional relationship between frailty and multimorbidity (i.e., the presence of two chronic conditions) [22]. In a cohort of 2722 individuals with human immunodeficiency virus (HIV), frailty positively and significantly predicted incident multimorbidity [23]. Meanwhile, there is strong evidence that multimorbidity is a risk factor for hospitalization in older adults [24]. Second, frailty is associated with cognitive decline [25], and individuals with cognitive decline are at an increased risk for hospitalization compared with those without cognitive decline [26]. Third, people with frailty may be more likely to be prescribed multiple drugs compared with their counterparts without frailty [27], and polypharmacy is significantly associated with referral to the hospital [28]. Fourth, frailty has been found to have deleterious effects on mental health [29], while impaired mental health is a risk factor for non-psychiatric hospitalization in old age [30].

### 4.3. Public Health Implications and Directions for Future Research

The study findings show that a substantial proportion of the older German population followed in general practices has more than three ICD-10 surrogates of the mFI, which is suggestive of frailty and is in line with previous studies conducted in Germany and using other frailty measures. In this context, measures preventing the occurrence of frailty in older adults living in the community are urgently warranted in Germany. These measures may include physical activity, oral health, and the management of non-communicable diseases [31]. Physical activity and exercise may act as preventive factors of frailty via their positive effects on muscle, pulmonary capacity, and cardiovascular function [32]. Sessions of physical activity may include different types of exercises, such as aerobic, balance, and strength exercises, and the content of these sessions may depend on the frailty status of the patient. In addition, multiple conditions (e.g., dementia, osteoarthritis, and Parkinson’s disease) and treatments (e.g., anticoagulants and bisphosphonates) may impair oral health in old age, and oral health advice should be regularly provided to older adults (e.g., brush teeth at least two times per day and remove dentures during the night) [33]. Regarding the management of non-communicable diseases, it should rely on integrated care, with improved coordination between healthcare professionals, better attention towards the coexistence of physical and psychiatric conditions, and increased awareness about potential inadequate polypharmacy [34]. Importantly, the measures mentioned above could buffer the deleterious impact of the ICD-10 surrogates of the mFI on referral to the hospital. Furthermore, the present study underlines the need for outpatient structures dedicated to the management and treatment of older people with frailty. Finally, in terms of future research, these surrogates may allow the study of the epidemiology of frailty and pre-frailty in primary care settings where diagnoses are coded using the ICD-10.

### 4.4. Strengths and Limitations

The major strengths of the study are the large sample size and the use of ICD-10 data routinely collected in primary care practices. Nonetheless, the study should also be interpreted in light of several limitations. First, although sarcopenia belonged to the 54 ICD-10 codes used to define the surrogates of the mFI in prior research [15], the ICD-10 code of sarcopenia (i.e., M62.84) was rarely documented in the practices included in the study, and the diagnosis of sarcopenia relied, therefore, on uncoded free text. In this context, the surrogate “functional status (not independent)”, which included sarcopenia, may have been underestimated. Second, there may have been some misdiagnoses, which may have biased the study results. Third, there was no data on health behaviors (e.g., alcohol consumption and physical activity). As some of these behaviors may be associated with both frailty and hospital referral, this lack of information may have partially confounded the Cox regression models. Fourth, the sample included older adults from general practices, and the findings cannot be generalized to those followed in specialized practices (e.g., internal medicine and geriatric practices) and those living in nursing homes.

## 5. Conclusions

The prevalence of more than three ICD-10 surrogates of the mFI (i.e., frailty) was relatively high in this older population followed in general practices in Germany. In addition, there was a positive and significant association between the number of ICD-10 surrogates of the mFI and 12-month referral to the hospital. These findings highlight the urgent need to better prevent the occurrence of frailty in general practices. Finally, more research based on ICD-10 data is warranted to investigate further the epidemiology of frailty in primary care settings.

## Figures and Tables

**Table 1:** ICD-10 surrogates of the modified Frailty Index.

Surrogate	ICD-10 Codes
Surrogate 1: “functional status (not independent)”	1. Visual impairment including blindness (binocular or monocular) (H54) 2. Abnormalities of gait and mobility (R26) 3. Other lack of coordination (R27) 4. Sarcopenia ^1^ 5. Age-related cognitive decline (R41.81) 6. Senility (R54) 7. Fracture of femur (S72) 8. Problems related to life-management difficulty (Z73) 9. Limitations of activities due to disability (Z73.6) 10. Problems related to care-provider dependency (Z74) 11. Need for assistance with personal care (Z74.1)
Surrogate 2: “history of hypertension requiring medication”	12. Essential (primary) hypertension (I10) 13. Hypertensive heart disease (I11) 14. Hypertensive renal disease (I12) 15. Hypertensive heart and renal disease (I13) 16. Secondary hypertension (I15)
Surrogate 3: “history of chronic obstructive pulmonary disease or pneumonia”	17. Viral pneumonia, not elsewhere classified (J12) 18. Pneumonia due to Streptococcus pneumoniae (J13) 19. Pneumonia due to Haemophilus influenzae (J14) 20. Bacterial pneumonia, not elsewhere classified (J15) 21. Pneumonia due to other infectious organisms, not elsewhere classified (J16) 22. Pneumonia in diseases classified elsewhere (J17) 23. Pneumonia, organism unspecified (J18) 24. Emphysema (J43) 25. Other chronic obstructive pulmonary disease (J44)
Surrogate 4: “history of impaired sensorium”	26. Creutzfeldt–Jakob disease (A81.0) 27. Dementia other than Alzheimer’s disease (F01 and F03) ^2^ 28. Organic amnesic syndrome, not induced by alcohol and other psychoactive substances (F04) 29. Delirium, not induced by alcohol and other psychoactive substances (F05) 30. Other mental disorders due to brain damage and dysfunction and to physical disease (F06) 31. Mental and behavioral disorders due to use of alcohol (F10) 32. Mental and behavioral disorders due to other substances (F11–F19) 33. Parkinson’s disease (G20) 34. Alzheimer’s disease (G30) 35. Other retinal disorders (H35)
Surrogate 5: “history of diabetes mellitus”	36. Type 1 diabetes mellitus (E10) 37. Type 2 diabetes mellitus (E11) 38. Other specified diabetes mellitus (E13) 39. Unspecified diabetes mellitus (E14)
Surrogate 6: “history of myocardial infarction”	40. Acute myocardial infarction (I21) 41. Subsequent myocardial infarction (I22) 42. Chronic ischemic heart disease (I25)
Surrogate 7: “history of congestive heart failure”	43. Heart failure (I50)
Surrogate 8: “history of stroke with neurologic deficit”	44. Intracerebral hemorrhage (I61) 45. Cerebral infarction (I63) 46. Sequelae of cerebrovascular disease (I69)
Surrogate 9: “history of TIA or stroke without neurological deficit”	47. Transient cerebral ischemic attacks and related syndromes (G45)
Surrogate 10: “history of PCI, angina or stenting”	48. Angina pectoris (I20)
Surrogate 11: “history of peripheral vascular disease or ischemic rest pain”	49. Atherosclerosis of arteries of extremities (I70.2) 50. Other peripheral vascular diseases (I73) 51. Stricture of artery (I77.1) 52. Disorder of arteries and arterioles, unspecified (I77.9)

Abbreviations: ICD-10, International Classification of Diseases-10th revision; TIA, transient ischemic attack; PCI, percutaneous coronary intervention. In the original study on which the present analyses are based [15], surrogate 7 also included chronic heart failure with the ICD-10 code U80.2. Given that chronic heart failure was not found in the ICD-10 manual used in this study, this diagnosis was not included in the analyses. ^1^ The diagnosis of sarcopenia relied on uncoded free text because the ICD-10 code of sarcopenia (i.e., M62.84) was rarely used. ^2^ Dementia other than Alzheimer’s disease was coded as F0 and F03 in one diagnosis and not as F00-F03 and F1 in two diagnoses as in the original study.

**Table 2:** Demographic characteristics of study patients.

Characteristic	N (%)
Age at the index date (in years)	
Mean (standard deviation)	77.0 (7.9)
65–69	311,069 (22.1)
70–74	281,557 (20.0)
75–79	262,588 (18.7)
80–84	279,103 (19.9)
85–89	177,851 (12.6)
≥90	93,870 (6.7)
Sex	
Women	790,263 (56.2)
Men	615,775 (43.8)

**Table 3:** Prevalence of the 11 ICD-10 surrogates of the modified Frailty Index in the overall sample.

	Total (N = 1,406,038)	Age 65–69 Years (N = 311,069)	Age 70–74 Years (N = 281,557)	Age 75–79 Years (N = 262,588)	Age 80–84 Years (N = 279,103)	Age 85–89 Years (N = 177,851)	Age ≥ 90 Years (N = 93,870)
Surrogate 1: “functional status (not independent)”	23.0	8.5	14.3	21.4	30.1	39.1	49.6
Surrogate 2: “history of hypertension requiring medication”	68.1	58.9	64.9	68.4	73.1	76.1	76.7
Surrogate 3: “history of chronic obstructive pulmonary disease or pneumonia”	18.2	15.5	16.6	18.0	19.4	21.6	22.5
Surrogate 4: “history of impaired sensorium”	21.1	13.8	14.7	18.3	24.3	33.0	40.3
Surrogate 5: “history of diabetes mellitus”	31.6	25.9	30.7	32.8	35.3	35.9	31.3
Surrogate 6: “history of myocardial infarction”	23.5	14.7	19.1	23.9	28.7	32.6	32.6
Surrogate 7: “history of congestive heart failure”	15.9	6.5	9.5	14.0	20.1	28.7	34.7
Surrogate 8: “history of stroke with neurologic deficit”	7.8	4.1	5.7	7.6	9.8	11.9	12.6
Surrogate 9: “history of TIA or stroke without neurological deficit”	3.3	1.8	2.4	3.2	4.1	5.1	5.6
Surrogate 10: “history of PCI, angina or stenting”	5.1	3.7	4.2	4.9	6.1	6.9	6.9
Surrogate 11: “history of peripheral vascular disease or ischemic rest pain”	9.2	6.3	7.9	9.5	11.0	11.9	11.8

Abbreviations: ICD-10, International Classification of Diseases-10th revision; TIA, transient ischemic attack; PCI, percutaneous coronary intervention.

**Table 4:** Association between the number of ICD-10 surrogates of the modified Frailty Index and 12-month incident hospital referral.

Group	0 ICD-10 Surrogate	1 ICD-10 Surrogate (HR [95% CI])	2–3 ICD-10 Surrogates (HR [95% CI])	>3 ICD-10 Surrogates (HR [95% CI])
Overall sample ^1^	Reference	1.37 (1.31–1.42)	2.00 (1.93–2.08)	3.32 (3.19–3.44)
By age (in years) ^2^				
65–69	Reference	1.36 (1.27–1.46)	1.99 (1.87–2.12)	3.48 (3.24–3.73)
70–74	Reference	1.33 (1.23–1.44)	1.93 (1.79–2.07)	3.45 (3.21–3.72)
75–79	Reference	1.37 (1.24–1.50)	1.93 (1.77–2.11)	3.39 (3.10–3.70)
80–84	Reference	1.33 (1.20–1.47)	1.96 (1.84–3.42)	3.11 (2.84–3.42)
85–89	Reference	1.48 (1.24–1.78)	2.20 (1.86–2.59)	3.39 (2.87–3.99)
≥90	Reference	1.59 (1.17–2.18)	2.57 (1.93–3.43)	3.51 (2.64–4.66)
By sex ^3^				
Women	Reference	1.43 (1.36–1.51)	2.15 (2.05–2.26)	3.42 (3.25–3.60)
Men	Reference	1.29 (1.21–1.37)	1.85 (1.75–1.95)	3.18 (3.01–3.37)

Abbreviations: ICD-10, International Classification of Diseases-10th revision; HR, hazard ratio; CI, confidence interval. All *p*-values are lower than 0.001. ^1^ Models adjusted for age and sex. ^2^ Models adjusted for sex. ^3^ Models adjusted for age.

## Data Availability

Data and code are available from the corresponding author upon reasonable request.

## Supplementary Materials

The following supporting information can be downloaded at: https://www.mdpi.com/article/10.3390/jcm12237290/s1, Table S1. Prevalence of the 11 ICD-10 surrogates of the modified Frailty Index in women. Table S2. Prevalence of the 11 ICD-10 surrogates of the modified Frailty Index in men.
