# Supplementary material for: International Classification of Diseases-10th Revision Surrogates of the Modified Frailty Index and 12-Month Referral to the Hospital in an Older Population from Germany
Source: J Clin Med. 2023 Nov 24;12(23):7290. doi: 10.3390/jcm12237290 (PMC10707624; doi:10.3390/jcm12237290)
Supplement: Supplementary file 1 [file jcm-12-07290-s001.zip › jcm-2655891-supplementary.pdf]

Supplementary Table S1. Prevalence of the 11 ICD-10 surrogates of the modified Frailty Index in women.

	**Total (N=790,263)**	**Age 65-69 years (N=162,412)**	**Age 70-74 years (N=148,580)**	**Age 75-79 years (N=142,113)**	**Age 80-84 years (N=159,168)**	**Age 85-89 years (N=111,274)**	**Age ≥90 years (N=66,716)**
Surrogate 1: "functional status (not independent)"	25.0	8.9	14.8	22.2	31.6	40.7	50.7
Surrogate 2: "history of hypertension requiring medication"	68.0	56.3	63.4	68.0	73.7	77.0	77.6
Surrogate 3: "history of chronic obstructive pulmonary disease or pneumonia"	16.5	14.1	15.0	16.0	17.2	19.3	20.3
Surrogate 4: "history of impaired sensorium"	20.8	11.2	12.5	16.9	24.0	33.8	41.7
Surrogate 5: "history of diabetes mellitus"	28.8	21.7	26.5	29.2	33.1	34.6	30.6
Surrogate 6: "history of myocardial infarction"	18.6	9.4	13.1	17.7	23.0	27.9	29.2
Surrogate 7: "history of congestive heart failure"	15.7	5.1	8.0	12.6	19.4	28.6	34.9
Surrogate 8: "history of stroke with neurologic deficit"	6.9	3.0	4.3	6.2	8.6	11.1	12.2
Surrogate 9: "history of TIA or stroke without neurological deficit"	3.2	1.6	2.1	2.9	3.9	4.8	5.5
Surrogate 10: "history of PCI, angina or stenting"	4.6	3.1	3.6	4.3	5.5	6.3	6.4
Surrogate 11: "history of peripheral vascular disease or ischemic rest pain"	7.3	4.3	5.6	7.1	8.8	10.0	10.6

Abbreviations: ICD-10, International Classification of Diseases-10th revision; TIA, transient ischemic attack; PCI, percutaneous coronary intervention.

Supplementary Table S2. Prevalence of the 11 ICD-10 surrogates of the modified Frailty Index in men.

	**Total (N=615,775)**	**Age 65-69 years (N=148,657)**	**Age 70-74 years (N=132,977)**	**Age 75-79 years (N=120,475)**	**Age 80-84 years (N=119,935)**	**Age 85-89 years (N=66,577)**	**Age ≥90 years (N=27,154)**
Surrogate 1: "functional status (not independent)"	20.4	8.2	13.8	20.3	28.1	36.3	46.7
Surrogate 2: "history of hypertension requiring medication"	68.2	61.8	66.6	68.9	72.4	74.5	74.4
Surrogate 3: "history of chronic obstructive pulmonary disease or pneumonia"	20.4	16.9	18.5	20.4	22.3	25.4	27.8
Surrogate 4: "history of impaired sensorium"	21.5	16.7	17.1	20.0	24.7	31.6	37.0
Surrogate 5: "history of diabetes mellitus"	35.2	30.5	35.3	37.0	38.2	38.0	33.1
Surrogate 6: "history of myocardial infarction"	29.9	20.6	25.7	31.3	36.1	40.5	40.9
Surrogate 7: "history of congestive heart failure"	16.2	8.0	11.2	15.6	21.1	28.8	34.4
Surrogate 8: "history of stroke with neurologic deficit"	8.9	5.4	7.1	9.2	11.3	13.3	13.6
Surrogate 9: "history of TIA or stroke without neurological deficit"	3.5	2.1	2.7	3.6	4.5	5.6	6.1
Surrogate 10: "history of PCI, angina or stenting"	5.8	4.4	4.9	5.6	6.8	7.9	8.1
Surrogate 11: "history of peripheral vascular disease or ischemic rest pain"	11.7	8.4	10.5	12.4	14.0	15.2	14.6

Abbreviations: ICD-10, International Classification of Diseases-10th revision; TIA, transient ischemic attack; PCI, percutaneous coronary intervention.
